# Maintenance of host DNA integrity in field-preserved mosquito (Diptera: Culicidae) blood meals for identification by DNA barcoding

**DOI:** 10.1186/s13071-016-1791-z

**Published:** 2016-09-15

**Authors:** Lawrence E. Reeves, Chris J. Holderman, Jennifer L. Gillett-Kaufman, Akito Y. Kawahara, Phillip E. Kaufman

**Affiliations:** 1 Entomology and Nematology Department, University of Florida, PO Box 110620, 1881 Natural Area Drive, Gainesville, FL 32611 USA; 2 Biosecurity Research Institute, Kansas State University, 1041 Pat Roberts Hall, Manhattan, KS 66506 USA; 3 McGuire Center for Lepidoptera and Biodiversity, Florida Museum of Natural History, University of Florida, 3215 Hull Road, Gainesville, FL 32611 USA

**Keywords:** *Aedes aegypti*, Blood meal preservation, Blood meal analysis, Blood meal identification, COI barcoding, Degraded DNA, DNA preservation, Host preference, Vector, Vector ecology

## Abstract

**Background:**

Determination of the interactions between hematophagous arthropods and their hosts is a necessary component to understanding the transmission dynamics of arthropod-vectored pathogens. Current molecular methods to identify hosts of blood-fed arthropods require the preservation of host DNA to serve as an amplification template. During transportation to the laboratory and storage prior to molecular analysis, genetic samples need to be protected from nucleases, and the degradation effects of hydrolysis, oxidation and radiation. Preservation of host DNA contained in field-collected blood-fed specimens has an additional caveat: suspension of the degradative effects of arthropod digestion on host DNA. Unless effective preservation methods are implemented promptly after blood-fed specimens are collected, host DNA will continue to degrade. Preservation methods vary in their efficacy, and need to be selected based on the logistical constraints of the research program.

**Methods:**

We compared four preservation methods (cold storage at -20 °C, desiccation, ethanol storage of intact mosquito specimens and crushed specimens on filter paper) for field storage of host DNA from blood-fed mosquitoes across a range of storage and post-feeding time periods. The efficacy of these techniques in maintaining host DNA integrity was evaluated using a polymerase chain reaction (PCR) to detect the presence of a sufficient concentration of intact host DNA templates for blood meal analysis. We applied a logistic regression model to assess the effects of preservation method, storage time and post-feeding time on the binomial response variable, amplification success.

**Results:**

Preservation method, storage time and post-feeding time all significantly impacted PCR amplification success. Filter papers and, to a lesser extent, 95 % ethanol, were the most effective methods for the maintenance of host DNA templates. Amplification success of host DNA preserved in cold storage at -20 °C and desiccation was poor.

**Conclusions:**

Our data suggest that, of the methods tested, host DNA template integrity was most stable when blood meals were preserved using filter papers. Filter paper preservation is effective over short- and long-term storage, while ethanol preservation is only suitable for short-term storage. Cold storage at -20 °C, and desiccation of blood meal specimens, even for short time periods, should be avoided.

## Background

The need to understand transmission system dynamics in insect-vectored pathogens has generated substantial interest in the taxonomic identification of vector blood meals [1]. Analysis of the blood meals of arthropods provides data necessary to understanding the epidemiology of arthropod-borne diseases. Techniques for blood meal identification have existed since the early twentieth century [2]. Some of the first methods for assigning taxonomic identities to blood meals were serological (e.g. enzyme-linked immunosorbent assays, precipitin tests) [3, 4]. Serological and early DNA-based methods were labor intensive, requiring the collection of serologic or genetic reference samples from each potential host taxon for laboratory comparison against blood meal specimens collected from arthropods in the field [1]. Because of this, it is difficult to screen blood meal samples against the entire range of potential hosts using these methods, and in some cases, host identifications are limited to higher taxonomic levels.

The use of mitochondrial DNA (mtDNA) and ribosomal DNA (rDNA) sequences to determine host species in contemporary blood meal identification methods improves the ability to identify a broader range of hosts to lower taxonomic levels [1]. Comparisons to reference samples remain necessary for taxonomic determinations of hosts. The rapid growth of DNA barcoding initiatives, such as Barcode of Life Data Systems (BOLD) [5], allows reference sample comparisons to be outsourced to publicly accessible sequence databases, without the need to independently collect and compare them to blood meals in the laboratory. In general, current methods are straightforward, and involve the extraction of host DNA from an arthropod, a PCR to amplify a diagnostic host DNA fragment, Sanger sequencing of the PCR product, and a database search for referenced sequences identical or similar to the blood meal-derived sequence. Several genetic markers have been used to identify hosts, but mtDNA is well suited to analyses of degraded DNA. Mitochondrial DNA is present in a high number of copies in many cells, is well conserved across animal taxa, and evolves rapidly, allowing even closely related taxa to be distinguished from one another. Regions of the cytochrome *c* oxidase subunit I (*cox1*) and cytochrome *b* (*cyt-b*) genes often are used because of their broad taxonomic coverage in databases.

DNA-based blood meal analyses require non-degraded fragments of vertebrate host DNA to serve as a template for amplification. Two major issues affect host DNA integrity and the subsequent success of PCR amplification of host DNA from blood meals: the extent of digestion [6] and DNA degradation during the preservation period prior to extraction [7]. Shortly after ingestion, imbibed blood meals are gradually digested and host DNA is degraded over time. Field samples of blood-fed arthropods consist of individuals containing blood meals at various stages of digestion [8]. Rates of DNA degradation due to digestion vary among hematophagous taxa. In mosquitoes, digestion proceeds quickly, with host DNA undetectable after 36–72 h [9, 10]. A similar rate of host DNA degradation is known from *Chrysomya* blowflies, with mammalian host DNA reliably detected up to 24 h after feeding, with detection dropping rapidly 48–96 h post-feeding [11]. In contrast, host DNA persists in leeches substantially longer, and can be amplified up to four months post-feeding [12]. Regardless of taxon, as post-feeding time increases, host DNA degrades and amplification becomes increasingly difficult. Unless effective preservation methods are implemented promptly after specimens are collected, downstream amplification of template DNA can be rendered impossible [13].

When blood-fed arthropods are field collected, subsequent preservation, transport, and storage can adversely affect the integrity of template DNA molecules, in part through the action of endogenous nucleases [14]. Preservation methods that are appropriate to the logistical constraints of a particular study program are required to optimize the ability to determine the taxonomic identity of host DNA. A variety of blood meal preservation methods have been used including chilling live arthropods during transport and prompt DNA extraction [9, 15], cold storage or cryopreservation until DNA extraction [16–19], desiccation of whole arthropod specimens [20, 21], blotting blood meals on filter paper for transport and storage [22, 23] and preservation of whole specimens in ethanol [24, 25]. When logistical constraints allow, the optimal method of preserving arthropod DNA is cryopreservation at -80 °C [26]. However, field logistics, particularly those of remote field sites or locations with limited access to freezers, can restrict the ability to utilize this method. Therefore, it is beneficial to identify preservation and storage methods appropriate to conducting blood meal identification research far from the laboratory or in the absence of access to ultra-low freezers.

In this study, we assessed the ability of four mosquito blood meal preservation methods to maintain template host DNA for subsequent analysis using the rapid and inexpensive HotSHOT DNA extraction method [27] in conjunction with a semi-nested primer set that selectively amplifies a 758 bp fragment of the *cox1* gene of a wide range of vertebrate classes (Mammalia, Aves, Reptilia, Amphibia) [28]. Although primer sets are available that target shorter fragments of bovine or mammalian mtDNA [21], we selected these primers for their versatility in targeting a wide range of potential host classes. This primer selection was made under the assumption that these primers are a logical choice for host identification from field-collected samples. The success of PCR amplification was used to assess the presence of sufficient concentrations of non-degraded template DNA for molecular analyses. The potential effects of storage time and post-feeding time on amplification success were investigated in our assessment of the performance of each preservation method.

## Methods

We experimentally compared the efficacy of four preservation methods (cold storage at -20 °C, silica desiccation, 95 % ethanol and filter papers) on the integrity of template host DNA in preserved mosquito blood meals. Maintenance of host DNA integrity during preservation was assessed by PCR amplification success. To evaluate the effects of storage and post-feeding time we preserved blood meal specimens at controlled intervals of post-feeding time points (every 6 h, from 6 to 54 h post-feeding), and held preserved specimens for a range of storage times (7, 30, 90 and 180 days). Our experimental procedures were replicated four times. Additionally, to confirm that the PCR worked properly with mosquito blood meals, and to estimate baseline PCR amplification success at the zero-hour post-feeding and 0 days of storage time point, we killed 16 blood-fed mosquitoes immediately following the two-hour feeding period. Four specimens were preserved using each preservation method, and DNA was extracted six hours after the feeding period ended.

### Mosquitoes

In each replication, approximately 700 laboratory-reared adult female *Aedes aegypti* (L.) (Diptera: Culicidae) (UF Strain, [29]) were removed from the colony maintained at the University of Florida, Veterinary Entomology Laboratory and held in a 30 × 30 × 30 cm wire-mesh cage, provisioned with one 14.8 ml vial of water with a paper wick, and one 7.4 ml vial of 10 % sugar solution with a cotton wick. All mosquitoes had eclosed approximately 7 days prior to removal from the colony, and had not taken a blood meal. Thereafter, two cotton pads (11 × 7 cm) were soaked in bovine blood until saturated and placed on top of the holding cage allowing mosquitoes to feed. After 2 h, individual mosquitoes were visually inspected for the presence of a blood meal. Approximately 200 blood-fed female mosquitoes were removed by aspiration, transferred to an identical cage and stored at 25 °C until preservation.

### Blood meal preservation

Within each replication, we preserved a sample of 144 blood-fed mosquitoes. Mosquitoes were preserved across nine serial time points, separated by six-hour intervals, from 6 to 54 h post-feeding. At each post-feeding time point, 16 mosquitoes were removed from the cage, and killed by exposure to ethyl acetate-soaked plaster in a 473 ml glass jar for 10 min. Of these, four individuals were preserved per preservation method described below, and placed into storage. From these four (per post-feeding time point), DNA was extracted from one mosquito blood meal at 7, 30, 90 and 180 days of storage. Altogether, this yielded a total sample of 576 mosquito blood meals (four preservation methods × 9 post-feeding time points × 4 storage times × 4 replications).

#### Desiccation

Prior to preservation, 1.5 ml graduated microcentrifuge tubes were prepared by filling to the 1 ml mark with silica beads (3.5 mm in diameter) (Consolidated Chemical and Solvents LLC, Quakertown, USA). A 3.5 × 3.5 cm square of crumpled Kimwipe^®^ tissue paper was inserted above the silica beads to prevent the mosquito specimen from directly contacting the silica. Specimens were preserved individually by placing the whole specimen on top of the tissue with sterile forceps. Tubes containing specimens were sealed for storage.

#### Cold storage

Whole mosquito specimens were placed individually into 1.5 ml microcentrifuge tubes. Within 10 min after death, specimens were transferred to, and stored in a -20 °C non-frost-free freezer until DNA extraction.

#### Ethanol

Whole mosquito specimens were transferred to 1.5 ml microcentrifuge tubes using sterile forceps. One ml of 95 % ethanol was added to each tube, and the tubes were sealed.

#### Filter paper

Using sterile forceps, mosquito specimens were individually transferred to the sampling area of a four-sample Flinders Technology Associates (FTA) card (Whatman^®^, Maidstone, United Kingdom). One mosquito was placed in each sampling area. The blood meal was released onto the sampling area by applying pressure with a sterile, plastic pestle. The pestle was then used to spread the blood meal around the sampling area until the card absorbed all viscous droplets. The card was air dried for 5 min before storage.

### Storage

Blood meal specimens preserved by desiccation, ethanol and filter paper were placed inside a cardboard box and stored inside an incubator at 30 °C and 80 % relative humidity. These settings were selected to simulate the conditions of field sites located in tropical or subtropical regions during the warmer months, under the assumption that preservation of specimens for molecular studies is most challenging under similar field conditions.

### DNA extraction

DNA was extracted from the blood meal specimens following the HotSHOT protocol [27]. Whole mosquito specimens preserved by desiccation, cold storage and ethanol were removed from storage and transferred to new 1.5 ml microcentrifuge tubes using sterile forceps. Seventy-five μl of lysis solution consisting of 25 mM NaOH and 0.2 mM EDTA were added to each tube. The abdomen of each specimen was individually macerated using a sterile pestle, releasing the blood meal into the solution. To avoid the release of endonucleases contained in the eyes, maceration of the thorax and head was avoided, and both were removed after the release of the blood meal. The lysis solution, containing homogenized blood meals, was transferred to 0.2 ml eight-well PCR strips.

Specimens preserved on filter paper were removed from storage and a 1 mm hole punch was used to remove 2 × 1 mm punch samples from the area containing the specimen residue. Using sterile forceps, the 2 punch samples per specimen were transferred to a 0.2 ml tube, and 75 μl of lysis solution was added to each well.

The eight-well strips, containing lysis solution and blood meals from all preservation methods, were incubated in a DNA Engine thermocycler (BioRad^®^, Hercules, USA) at 95 °C for 30 min followed by 4 °C for 5 min. Seventy-five μl of neutralization buffer consisting of 40 mM tris-HCL was added to each tube. The tubes were briefly vortexed and immediately frozen at -20 °C until PCR amplification. Polymerase chain reactions were performed within ten days of DNA extraction, and each sample underwent one freeze-thaw cycle (samples were frozen immediately after DNA extraction, and thawed prior to PCR).

### PCR

The presence of a sufficient concentration of non-degraded template DNA for PCR amplification was determined through a semi-nested PCR using primer pairs designed by Alcaide et al. [28]. The expected product of this reaction is a 758 bp fragment of the vertebrate *cox1* gene. The degenerated primers M13BCV-FW (5′-TGT AAA ACG ACG GCC AGT HAA YCA YAA RGA YAT YGG-3′) and BCV-RV1 (5′-GCY CAN ACY ATN CCY ATR TA-3′) were used in the first reaction. BCV-FW1 contains a M13 tail at the 5′ end to facilitate the second, semi-nested amplification reaction and sequencing. The M13 primer (5′-GTA AAA CGA CGG CCA CTG-3′) and the reverse primer BCV-RV2 (5′-ACY ATN CCY ATR TAN CCR AAN GG-3′) were used in the semi-nested reaction. Although primer pairs targeting shorter amplicons are preferred in blood meal analyses, the Alcaide [28] sets were used because they putatively amplify DNA templates from a wide range of vertebrate groups.

A BioRad® DNA Engine thermocycler was used in all reactions. Our semi-nested reactions followed a protocol slightly modified from Alcaide et al. [28]. The first reaction was performed in a final volume of 15 μl that contained 1 U of Taq polymerase (Sigma-Aldrich, St. Louis, USA), 1 × PCR buffer (Sigma-Aldrich), 2.5 mM MgCl_2_, 0.25 mM of each dNTP, 5 % DMSO, 10 μg of bovine serum albumin, 0.13 μM of each primer (M13BCV-FW1, BCV-RV1), and 1 μl of DNA extract. The first amplification reaction consisted of an initial denaturation step of 94 °C for 4 min, followed by 35 cycles of 45 °C for 40 s, 72 °C for 1 min and 94 °C for 4 min, with a final extension step of 72 °C for 7 min. The second reaction was performed in a final volume of 15 μl that contained 1 unit of Taq polymerase (Sigma-Aldrich), 1× PCR buffer, 1.7 mM MgCl_2_, 0.25 mM of each dNTP, 5 % DMSO, 5 μg bovine serum albumin, 0.16 μM of each primer (M13, BCV-RV2), and 0.5 μl of PCR product from the previous reaction. The second reaction consisted of an initial denaturation step of 94 °C for 3 min, followed by 16 cycles of a touchdown program of 60 °C to 45 °C for 40 s, 72 °C for 1 min, and 94 °C for 40 s. During these 16 cycles, the annealing temperature began at 60 °C and was decreased by 1 °C per cycle to 45 °C on the 16th cycle. The touchdown cycles were followed by 24 cycles of 94 °C for 40 s, 45 °C for 40 s and 72 °C for 40 s, with a final extension step of 72 °C for 7 min. Negative controls containing no DNA were used in every set of reactions to monitor for contamination.

The products of the semi-nested reaction were electrophoresed and visualized on an ethidium bromide-stained 1.5 % agarose gel. The presence of a band at the expected amplicon size was interpreted as a positive result, indicating sufficient host DNA preservation for blood meal identification. We verified amplification of the correct vertebrate template using Sanger sequencing on an ABI 3130® automated sequencer at the University of Florida Interdisciplinary Center for Biotechnology Research (ICBR). A subset of PCR products from successful amplification reactions were sequenced, and the resulting sequences were searched in the National Center for Biotechnology Information (NCBI) sequence database using the Standard Nucleotide Basic Local Alignment Search Tool.

### Statistical analysis

Data were analyzed in the statistical program R® Version 3.2.0 using the stats, *lsmeans*, and *multcompView* packages [30]. We modeled the effects of the independent variables, preservation method, storage time and post-feeding time, on the dependent variable, amplification success, using a multivariate analysis, binomial logistic regression. Because direct comparisons of independent variable coefficients can be misleading in logistic regression models [31], we used equality tests of predicted probabilities to identify differences in amplification success [32]. We applied this approach to our data to test for amplification success differences among the four preservation methods with the continuous variables held at a range of constant values.

An initial model included all independent variables and their interactions. Using a backward stepwise elimination process, we selected the terms of the final model that best explained data variability based on the Akaike information criterion [33]. Model fit was evaluated with the Hosmer-Lemeshow goodness-of-fit test, and the likelihood ratio test. We evaluated the significance of the overall effect of each variable coefficient in the model using the Wald Chi-squared statistic. Least squares means (LS means) were used to conduct pairwise comparisons of the predicted probability of amplification for each preservation method. Because amplification success varies with the levels of the continuous variables, pairwise comparisons were made with storage time and post-feeding time held at constant values. For this analysis, we selected 7, 77 and 180 days, and 6, 30 and 54 h as constant values of storage time and post-feeding time, respectively, to represent brief, intermediate and extended values of each variable. Pairwise comparisons were made on the LS means of the predicted probability at all combinations of these values, and all comparisons were made at the 95 % confidence level.

## Results

We verified that the PCR procedure amplified the correct DNA template, and amplification success was 100 % for blood meal specimens preserved at and stored for 0 h post-feeding. All sequenced PCR products contained DNA fragments that matched the *cox1* sequence of the expected host species, with > 99 % similarity to databased *Bos taurus cox1* sequences.

**Fig. 1:**
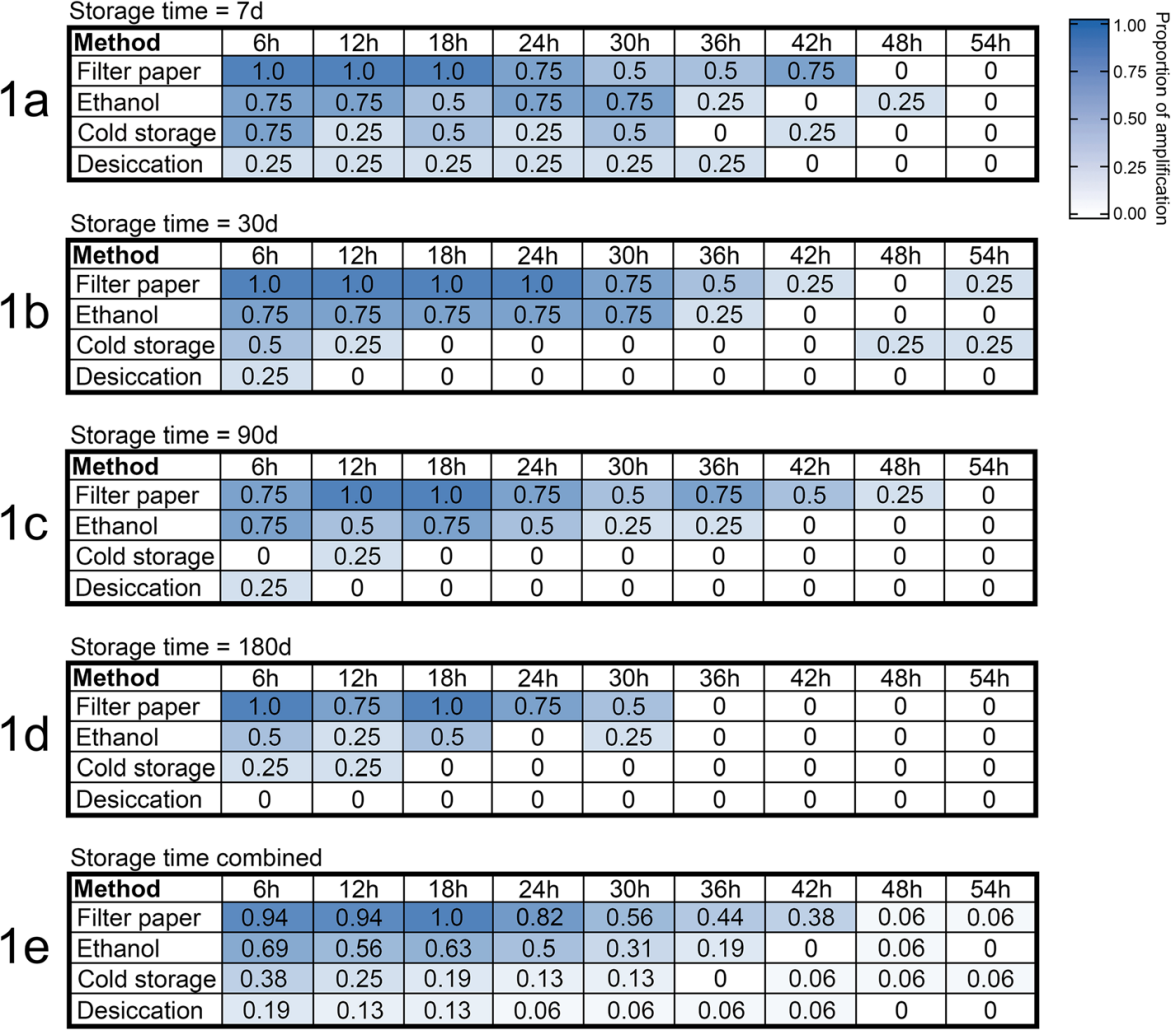
The observed proportion of amplification success by preservation method at each storage time (**a**–**e**), and post-feeding time. Blood meal specimens were stored for seven days (**a**), 30 days (**b**), 90 days (**c**) and 180 days (**d**). For each preservation method, storage time and post feeding time treatment combination, a total of four blood meal specimens were tested. The bottom sub-figure (**e**) presents the proportion of amplification success for each treatment across storage times (*n* = 16 for each preservation method and post-feeding time combination). Cell shading corresponds with the observed proportion of amplification success for each treatment, with blue indicating a proportion of 1.0, and white indicating zero

Host DNA templates were amplified from specimens preserved by each of the 4 preservation methods. Altogether, 159 of the 576 blood meal specimens contained host DNA that could be detected by our PCR. Amplification success was greatest in specimens preserved on filter papers, and in ethanol (Fig. 1). Amplification success was poor for specimens preserved through cold storage, and desiccation. Overall, storage time and post-feeding time negatively affected amplification success, and for all preservation methods, the proportion of successful amplification was greatest at earlier storage and post-feeding times.

In the final logistic regression model, the effects of preservation method, storage time, post-feeding time and the interactions between preservation method and storage time, and preservation and post-feeding time were the model predictors that minimized the Akaike information criterion. According to the model, storage time and post-feeding time had significant effects, and were negatively related to amplification success (*P* < 0.0001 for both). Wald Chi-squared tests on the coefficient estimates for each predictor indicated that all but the interaction between preservation method and storage time significantly contributed to model fit, although the inclusion of this variable in the final model minimized the Akaike information criterion (Table 1). The Hosmer-Lemeshow goodness-of-fit test indicated that the model fit the data well, and there was not a significant difference between the model and the observed data. The likelihood ratio test compared the full model against a null model, and indicated that the full model had a significantly better fit (*χ*^2^ = 256.1, *df* = 5, *P* < 0.0001).

**Table 1:** The best-fit logistic regression model predicting amplification success for four host DNA preservation methods

	Wald *χ* ^2^	*df*	*P*
Intercept	20.4	1	< 0.001
Preservation method	24.8	3	< 0.001
Post-feeding time	28.5	1	< 0.001
Storage time	10.9	1	< 0.001
Preservation method × post-feeding time	10.9	3	0.012
Preservation method × storage time	5.9	3	0.12

The dependent variable in this analysis was amplification success, coded so that 0 = no amplification, and 1 = amplification. The inclusion of the selected variables important in predicting PCR amplification success was based on comparisons of the Akaike information criterion. The significance of individual variables in predicting amplification success was determined by Wald Chi-squared tests at the 95 % confidence interval. Model fit was assessed with the Hosmer-Lemeshow goodness-of-fit test (*χ* ^2^ = 6.81, *df* = 8, *P* = 0.557), and likelihood ratio test (*χ* ^2^ = 256.1, *df* = 5, *P* < 0.001). The best-fit model was identified as logit(amplification success) = 2.608–2.146 cold–2.297 desiccation + 2.208 filter paper–0.012 storage–0.089 post-feeding–0.085 cold*storage–0.004 desiccation*storage + 0.006 filter paper*storage + 0.044 cold*post-feeding + 0.041 desiccation*post-feeding–0.04 filter paper*post-feeding. In the model, preservation method is a categorical variable, dummy-coded with ethanol as the reference level

**Fig. 2:**
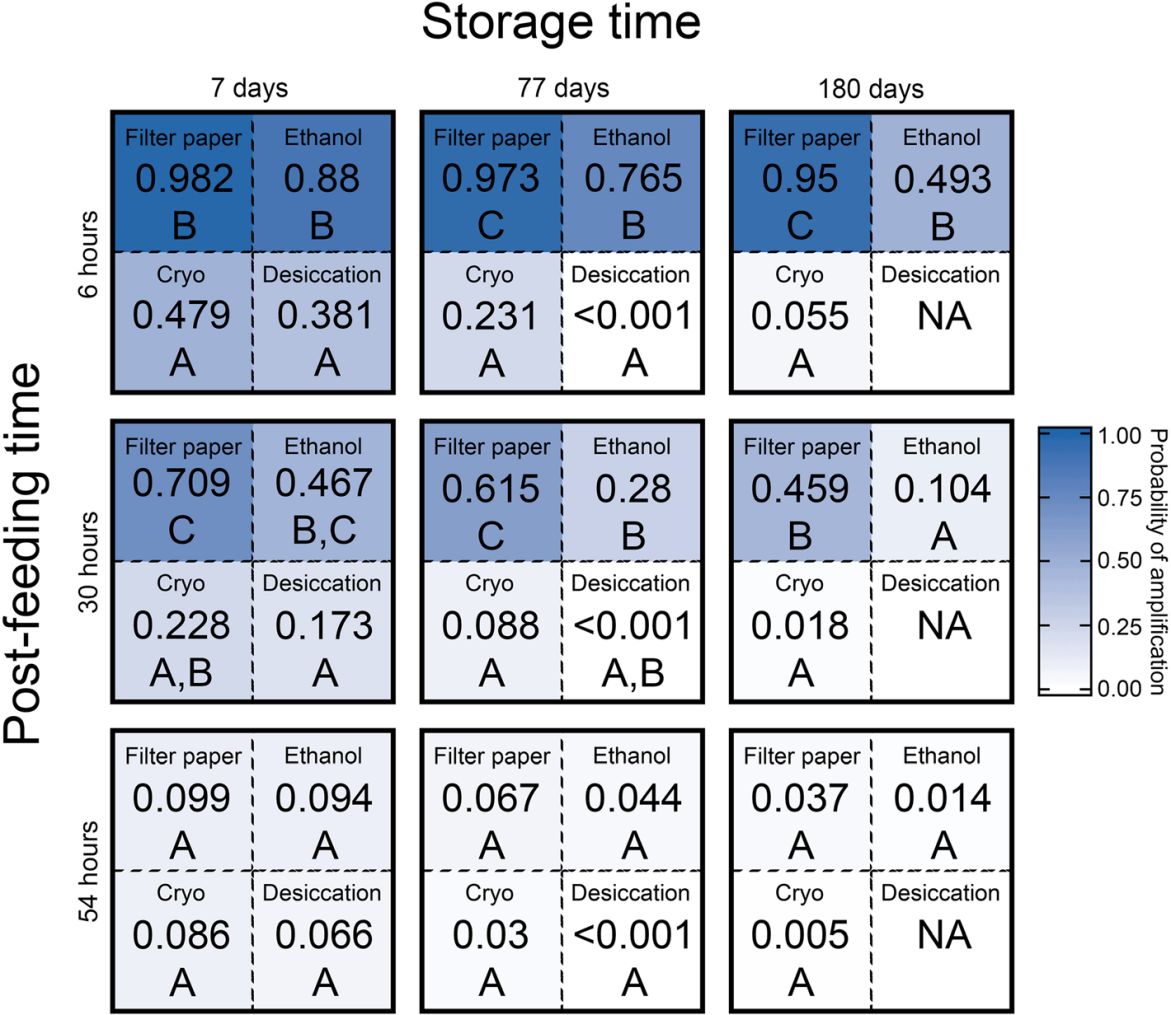
Pairwise LS means comparisons of the predicted probability of amplification success of the four preservation methods. Comparisons were made with storage time and post-feeding time held at a range of constant levels, indicated on the axes. Each of the nine large boxes represents one pairwise comparison of all preservation methods. Numerical values inside each dotted-line box indicate the LS mean of the predicted probability of amplification success for the specified preservation method. Preservation method LS means with the same letters are not significantly different, and means with different letters are significantly different, both at the 95 % level of confidence. The shading of dotted-line boxes is scaled to the LS mean of the predicted probability of amplification success, with blue indicating a predicted probability of 1.0, and white indicating a predicted probability of zero

No blood meals from desiccation-preserved samples stored for 180 days amplified, preventing pairwise comparisons among treatments at this storage time. For this reason, desiccation was excluded from pairwise comparisons at 180 days of storage. Overall, the predicted probability of amplification decreased as storage time and post-feeding time increased. With storage time and post-feeding time at their minimums (7 days and 6 h, respectively), the LS means were highest in all preservation methods (Fig. 2). Throughout the range of storage and post-feeding values, LS means for cold storage and desiccation were low (<0.5), and no significant differences were found between these preservation methods (*P* > 0.2357).

Filter paper preservation had more instances of amplification success than any other method. The predicted probability of amplification for filter paper was consistently higher than all other preservation methods, and LS means were significantly different than all other methods at 77 (*P* < 0.003) and 180 (*P* < 0.009) days of storage, with post-feeding time at 6 and 30 h. After storage for 77 and 180 days, the predicted probability of amplification for filter paper-preserved specimens remained high (> 0.9) with post-feeding time at 6 h, while for ethanol the predicted probability decreased from ~ 0.9 at 7 days of storage, to ~ 0.5 at 180 days. At 7 days of storage, ethanol was not significantly different from filter paper preservation at 6 and 30 h post-feeding (*P* > 0.0767). Together, these results indicate that at 7 days of storage, filter papers and ethanol maintained host DNA integrity equally well. At extended storage times, filter paper preservation outperforms all other methods. With post-feeding time at 54 h, predicted probabilities for all methods were < 0.1, and there were no significant differences between preservation methods (*P* > 0.1909).

## Discussion

This study demonstrates that preservation method affects amplification success, and thus the effectiveness of hematophagous invertebrate host identification. In molecular analyses of field-collected specimens, the maintenance of DNA integrity should be considered when selecting the appropriate specimen preservation methods [34].

When host DNA contained in the blood meal of a hematophagous invertebrate is the target of molecular analysis, storage-associated degradation needs to be prevented, and digestive processes must to be suspended. We found that filter paper preservation had the highest predicted probability of amplification success across all storage and post-feeding times. At extended periods of storage, the predicted probability for successful filter paper preservation was significantly greater than the other methods (*P* < 0.009). As we attempted to mimic tropical field conditions, with respect to temperature and humidity, as well as likely storage periods between collection and extraction, our results suggest that filter paper preservation is a suitable method under such challenging conditions. However, compared with the other preservation methods we investigated, filter paper preservation is the most costly. A package of 100 four-sample Whatman^®^ FTA Blood Cards costs ~ $500, while the cost of the other preservation methods is negligible. Ethanol preservation was less effective for long-term preservation of host DNA than filter papers, but was otherwise the most suitable method.

All preservation methods evaluated were found to be sensitive to increasing storage and post-feeding time. The negative effect of post-feeding time was expected because enzymatic activity in the mosquito digestive system begins to degrade host DNA immediately after ingestion [35]. Prior to preservation, accumulation of enzymatic degradation decreases the concentration of intact template host DNA molecules, reducing the probability of amplification [36]. Our data reflect this trend, with the predicted probability of amplification success for all methods consistently decreasing as post-feeding time increased. By 42 h after feeding, PCR amplification was unsuccessful in the majority of instances. At 54 h post-feeding, the predicted probability of amplification success was < 0.1 for all methods, and no significant differences were found between the preservation methods. This result corresponds to the findings of previous studies [9, 10, 37] that report difficulty in amplifying host DNA 36–72 h following ingestion.

Samples of field-collected blood-fed mosquitoes consist of individuals containing blood meals of various ages [8]. Unless mosquitoes are collected directly from the host, it is difficult to reliably estimate the post-feeding time of sampled mosquitoes collected through standard techniques (e.g. resting shelter traps, collection at natural resting sites) [38]. Loss of host DNA template molecules to digestive degradation is inevitable in field-collected samples. Upon collection, it then becomes important to promptly suspend the effects of digestion, and protect remaining host DNA templates from further degradation through the implementation of an effective preservation method. We found that the interaction between preservation method and post-feeding time affects amplification success, suggesting that there are differences between preservation methods in terms of their ability to prevent further degradation. Effective preservation methods also need to protect template DNA from storage-associated degradation caused by oxidation, hydrolysis, or radiation [34]. Storage time had a significant negative effect on the predicted probability of amplification success (*P* < 0.0001). For all preservation methods, predicted probability of amplification decreased with increasing storage time, suggesting that none of the methods entirely protected host DNA integrity.

Investigations of the host-use of mosquitoes and other hematophagous invertebrates sometimes take place at remote field sites, necessitating field storage of specimens prior to laboratory processing. Of the methods we tested, only filter papers and ethanol can be considered suitable field storage methods for blood meals sampled from remote sites. Both are viable methods for short-term field storage of blood meals. This result is in agreement with previous findings [37] that suggest filter papers and ethanol are effective preservation media for mosquito blood meals. Considering that the Whatman® FTA brand of filter papers used here have preserved blood samples that yield amplifiable DNA after at least 8.5 years, and may remove PCR inhibitors from the sample [39], it is not surprising that this method largely outperformed the others. Whatman® FTA Blood Cards have relevant applications beyond blood meal preservation. Viral RNA can be preserved, and stored for years, on these cards [40, 41], making it possible to preserve both vector DNA and pathogen DNA or RNA [23].

The selection of blood meal preservation methods for field storage and transportation should be determined by the constraints of fieldwork [34], including available facilities and equipment, duration of the trip, remoteness of field sites and permits needed for domestic and international transportation (Table 2). Neither filter papers nor ethanol require specialized equipment, and with both, specimens can be stored at ambient temperatures. Of the two, filter paper preservation is the most versatile in terms of long-term stability of host DNA, and ease of transport. Comparatively, ethanol preservation is best suited to short-term storage applications, and airline or governmental regulations may make it difficult to transport ethanol to field sites.

**Table 2:** Comparison of storage methods, and factors important to the selection of the appropriate method

Storage method	Suitability for short-term field storage	Suitability for long-term field storage	Cost	Operational concerns	Additional requirements	Conclusions
Filter paper	Excellent	Excellent	Comparatively expensive: ~$500 per pack of 100	Cards holding DNA specimens require careful handling to protect from contamination during storage and transport.	Supply of sterile instruments or ability to sterilize instruments used to macerate insect abdomen onto card.	Effective for short- and long-term specimen storage. Substantial cost is a disadvantage.
EtOH	Excellent	Suitable	Negligible: < $5 per liter	Airline restrictions on ethanol in baggage, and transporting ethanol internationally.	Tubes, or other containers to individualize blood meal specimens.	Effective for short-term storage, and inexpensive, but potential transportation issues.
-20 °C	Poor	Poor	Negligible	Continued preservation required during transit. Availability of equipment.	Access to electricity for freezers, or supply of dry ice or liquid nitrogen.	Cold storage at -20 °C should be avoided, even when field conditions permit its use.
Silica	Poor	Poor	Negligible	Airtight containers needed to protect silica from moisture absorption.	Tubes, or other containers to individualize blood meal specimens.	Preservation of blood meal specimens is ineffective. The method should be avoided.

In our study, cold storage at -20 °C and desiccation were the least effective methods for preserving host DNA. We estimated the baseline (zero hours post-feeding time, zero-hours storage) probability of amplification to be 1.0. At the earliest storage and post-feeding values (seven days, and six hours, respectively) included in the model, the LS means estimates of the predicted probability of amplification for cold storage and desiccation were 0.479 and 0.381, respectively. The decrease in the predicted probability of amplification success from 1.0 at zero hours/zero days to < 0.5 at six hours/seven days indicates that host DNA degradation occurs rapidly in cold-stored and desiccated blood meal specimens. This finding is particularly concerning given the widespread use of cold storage of genetic samples prior to molecular analysis.

Cold storage at -20 °C is often a convenient method used to store blood meal samples prior to molecular analyses [6, 10, 18, 42]. Our results suggest that storage of specimens at -20 °C, even for short time periods, is detrimental to the effectiveness of downstream host DNA amplification. In similar molecular analyses of the gut contents of predators, Passmore [43] found that 80 % ethanol preserved prey DNA better than cold storage at -20 °C. Frost-free -20 °C freezers are often available at biological stations and other field accommodations when sampling takes place far from the laboratory. Frost-free freezers do not maintain a constant -20 °C. Rather, the temperature fluctuates, and the freezers periodically thaw to prevent ice buildup via dehumidification of water vapor. Our use of a freezer that maintained specimens at a constant -20 °C resulted in poor amplification success. The temperature fluctuations of frost-free freezers are believed to accelerate DNA degradation [44]. When available, the use of -20 °C freezers at field sites presents further challenges in that the temperature needs to be maintained during transport back to the laboratory. Although blood meal specimens are typically held at -70 or -80 °C in the laboratory, such equipment is often unavailable in remote field sites, leaving researchers in search of methods that effectively preserve DNA at ambient temperatures.

Desiccation of blood meal specimens does not require cold temperatures, but our results suggest preservation of mosquito blood meals through desiccation can be problematic. This result aligns with a previous study that reported PCR amplification to be rare in silica-preserved mosquito blood meal specimens [20]. However, Kent and Norris [21], and Logue et al. [45] successfully used silica to preserve mosquito blood meals collected from remote field sites. Kent and Norris [21] used a species-specific multiplexed PCR that amplifies a 132–680 bp fragment, depending on host species, of the mammalian *cyt-b* gene. Logue et al. [45] used high throughput (Illumina) sequencing of a 140 bp fragment of the mammalian 16S ribosomal RNA gene. High throughput sequencing and primer sets targeting shorter amplicons are well suited for analyses of degraded host DNA. The use of such molecular methods that are better suited to degraded DNA templates may be compatible with desiccation-preserved blood meals. However, the results presented here suggest that preservation of blood meals by desiccation should be avoided. Sanger sequencing and primers targeting longer template fragments are used. In addition, desiccation by drying insects with silica is successfully used to preserve insect DNA for molecular analyses [46, 47]. We suspect that when host DNA, rather than insect DNA, is the target of molecular analysis, desiccation of blood-fed mosquito specimens with silica gel is not accomplished quickly enough to adequately block the action of digestive enzymes.

In addition to the variables we tested, amplification success of host DNA can be affected by the method used to kill invertebrates [48], the method used to extract DNA [26, 34] and the length of the amplicon [22, 49]. Martinez-de la Puente et al. [36] found amplification success improved by 17 % when DNA was extracted using commercial Qiagen® DNA extraction kits as compared with the HotSHOT DNA extraction method [27]. However, the cost per sample of using commercial DNA extraction kits is substantial in comparison to the HotSHOT method. Amplicon length also can affect the probability of amplification success.

Molecular analyses that require the amplification of highly degraded DNA (e.g. environmental DNA, ancient DNA, DNA from old museum specimens) often target short amplicons 80–250 bp in length [50]. The number of strand breaks in individual DNA molecules increases with time, resulting in DNA degradation, thereby decreasing the number of copies of intact DNA molecules available to serve as templates. As a result, intact copies of 100 bp DNA templates are likely to persist longer than 1,000 bp DNA templates, and amplification is more likely to be successful with shorter amplicons [51]. Currently, no barcoding primer sets have been published for shorter amplicons in the ~100 bp size range that target a diverse set of vertebrate classes, while excluding amplification of insect templates.

Universal barcoding primers are available to amplify shorter fragments, however these co-amplify invertebrate and host DNA. Such an approach requires high throughput sequencing, coupled with a bioinformatics platform to parse amplified invertebrate DNA fragments from those of hosts. Host identification through high throughput sequencing can be advantageous because the use of universal primer pairs targeting shorter templates makes the method more sensitive to degraded DNA, and by making identification of mixed blood meals, derived from more than one host, tractable. These results should be equally applicable to research that uses a high throughput sequencing approach.

## Conclusions

In this study we demonstrate that preservation method affects the success of PCR amplification of host DNA contained in mosquito blood meals. When sampling mosquito populations that are located in close proximity to the laboratory, preservation of blood meal specimens is relatively straightforward. When blood-fed invertebrates are collected from remote locations, maintenance of host DNA integrity during field storage can be problematic. Here, we show that all the preservation methods we tested were sensitive to storage time and post-feeding time. It is impossible to correct DNA degradation due to digestion through a preservative. However, because host DNA is inherently degraded when it is placed in storage, it is important that the preservation method block continued enzymatic digestion, as well as degradation due to oxidation, hydrolysis and radiation. Our results strongly suggest that filter paper preservation is the most reliable and versatile of the methods we tested. At extended periods of storage, filter paper preservation outperformed all other methods. Ethanol preservation was not significantly different from filter paper preservation over short-term periods of storage. In general, cold storage at -20 °C and desiccation performed poorly and neither method is a viable technique for preserving mosquito blood meals collected at remote field sites. Cold storage is a widespread method for preserving DNA integrity, and -80 °C is thought to be an acceptable method for storing host DNA. Future work should examine these variables further to determine the threshold at which cold storage is ineffective.
